# Predicting implementation of the PAX Good Behavior Game + MyTeachingPartner interventions

**DOI:** 10.3389/fpsyg.2023.1059138

**Published:** 2023-03-10

**Authors:** Summer S. Braun, Catherine P. Bradshaw, Lydia A. Beahm, Alexa C. Budavari, Jason Downer, Nicholas S. Ialongo, Patrick H. Tolan

**Affiliations:** ^1^Department of Psychology and Center for Youth Development and Intervention, University of Alabama, Tuscaloosa, AL, United States; ^2^School of Education and Human Development, University of Virginia, Charlottesville, VA, United States; ^3^Bloomberg School of Public Health, Johns Hopkins University, Baltimore, MD, United States

**Keywords:** implementation science, good behavior game, feasibility, teacher well-being, occupational health

## Abstract

**Introduction:**

Effective classroom management is critical to creating a classroom environment in which social, emotional, and academic learning can take place. The present study investigated the association between early career, early elementary teachers’ occupational health (job stress, burnout, and perceived teaching ability) and perceptions of program feasibility in relation to their implementation dosage and quality of two evidence-based classroom management programs implemented together: the PAX Good Behavior Game (GBG) and MyTeachingPartner (MTP) intervention.

**Methods:**

Teachers provided information on their occupational health at the start of the school year and were then randomized to the PAX GBG + MTP condition or control condition. Teachers’ perceptions of the feasibility of the program, implementation dosage, and implementation quality of the intervention were measured at the end of the school year for the 94 intervention teachers.

**Results:**

Teachers participated in more MTP coaching cycles when they reported that the combined PAX GBG + MTP program was feasible. Although there were no main effects of occupational health on implementation, the associations between job stress and implementation quality were moderated by perceptions of feasibility.

**Discussion:**

Findings highlight the complexity of factors influencing the implementation of evidence-based programs in school settings.

## 1. Introduction

Social and emotional learning programs and classroom management interventions are known to improve children’s social and emotional skills, attitudes, behavior, and academic performance (Durlak et al., 2011; Korpershoek et al., 2016). Yet, effective implementation of such evidence-based intervention programs is critical to producing these targeted outcomes (Kam et al., 2003; Durlak and DuPre, 2008; Domitrovich et al., 2010). Schools are inherently complex systems, and there is noted variation in the implementation of such programs within this context (Witt et al., 1997; Ringwalt et al., 2009; Sanetti and Kratochwill, 2009; Hicks et al., 2014; Sanetti and Collier-Meek, 2019). To advance the science of implementation, it is essential to understand the factors influencing the adoption of evidence-based programs. The present study aimed to contribute to the practical understanding of implementation in the school context by exploring the effects of teachers’ occupational health and their perceptions of feasibility of two evidenced-based interventions employed together: PAX Good Behavior Game (GBG) and MyTeachingPartner (MTP).

### 1.1. Importance of evidence-based programs for youth

Evidence-based programs are those which have undergone rigorous scientific testing and are found to have beneficial effects for the target population (What Works Clearinghouse, 2017). Evidence-based social and emotional learning programs are known to have widespread positive effects on children: improving social and emotional skills, attitudes towards self and others, positive social behavior, mental health, and academic performance, as well as preventing conduct problems and emotional distress (Durlak et al., 2011). Yet, effective classroom management is critical to creating a classroom environment where such learning can occur (Evertson and Weinstein, 2011). In fact, classroom management interventions themselves have been shown to benefit not only classroom behavior but also students’ academic outcomes and social and emotional development (Korpershoek et al., 2016). Further, evidence-based social and emotional learning and classroom management interventions can have effects long after program participation, even in areas not directly targeted by the intervention (e.g., graduation; Taylor et al., 2017). Based on evidence that early-career teachers struggle specifically with classroom management (Kukla-Acevedo, 2009; Wei et al., 2010), which is foundational to student learning, this project combined two interventions with a strong evidence-base for improving classroom management: a classroom management program, the PAX GBG, and a teacher coaching intervention, MTP.

#### 1.1.1. PAX GBG

The GBG is an evidence-based program originally developed by Barrish et al. (1969) that aims to promote teachers’ classroom behavior management, increase on-task behaviors, and decrease disruptive behaviors (Bowman-Perrott et al., 2016; Smith et al., 2021). The GBG incorporates several behavior management strategies such as positive behavior praise, explicit instruction, feedback, and positive reinforcement, thus making it a high-quality practice for teachers to implement in their classrooms. The GBG is an interdependent group contingency that requires all students in the group to meet the requirements of the contingency as a group and individually (Embry, 2002). As such, students typically encourage their peers to meet expectations, thus reducing some of the demands on teachers (Hopman et al., 2018). GBG includes identifying the target behavior (e.g., completing the worksheet silently), posting the GBG expectations, dividing the class into equal teams, and awarding points to teams meeting expectations or removing points for infractions (Barrish et al., 1969; Embry, 2002). The team with the most points receives a non-tangible group reinforcement. The PAX GBG augmented the original version of GBG by integrating additional activities and components to improve compliance and classroom management (e.g., soliciting student input on classroom expectations; Embry, 2002). Previous research has demonstrated positive effects of the GBG and the PAX GBG for both students (e.g., reduced aggressive/disruptive behaviors and improved academic outcomes; Ialongo et al., 2019; Johansson et al., 2020; Smith et al., 2021; Leidig et al., 2022) and teachers (e.g., decreased emotional exhaustion; Hopman et al., 2018).

#### 1.1.2. MTP

MTP is an evidence-based coaching intervention that targets effective classroom management through the quality of teachers’ interactions with students (Allen et al., 2015). Throughout the program, coaches provide video-based, individualized feedback to teachers as they develop classroom management skills and the capacity to provide emotional and instructional support to students. Previous studies of MTP have demonstrated positive effects on the quality of student-teacher interactions, peer interactions, social functioning, behavioral engagement, and academic outcomes (Pianta et al., 2008b; Allen et al., 2011, 2015; Mikami et al., 2011; Gregory et al., 2014).

Given the evidence behind the PAX GBG and MTP, recent research has combined both programs in an effort to support the development of early-career teachers’ classroom management skills and capacity for high-quality interactions with students (Tolan et al., 2020). In this combined approach, the PAX GBG and MTP work in tandem, incorporating unique and overlapping classroom management strategies that aim to improve teachers’ interactions with students, their classroom management practices, and subsequent student outcomes. Early-career teachers are a particularly suitable population for these interventions because, in comparison to more experienced teachers, they are actively developing new habits, may be more open to feedback, and may need new classroom management skills, given that many pre-service preparation programs are known to provide insufficient training in this area (Freeman et al., 2014).

### 1.2. Importance of implementation

Importantly, effective implementation of evidence-based programs such as the PAX GBG and MTP is critical to their success. A meta-analysis of 542 studies of interventions for youth concluded that implementation had profound effects on outcomes; programs implemented well resulted in effect sizes several times higher than those with poorer implementation (Durlak and DuPre, 2008). Despite their potential convenience for improving public health, school-based interventions are often at risk for poor implementation (Domitrovich et al., 2008; Sanetti et al., 2014). The emergence of the field of implementation science has brought an explicit focus on understanding and in turn, addressing the barriers that jeopardize the effective implementation of such programs (Eccles and Mittman, 2006; Durlak, 2015).

### 1.3. Conceptualization of implementation

Implementation refers to the content of the program and how it is delivered in a specific setting (Durlak and DuPre, 2008). Durlak and Dupre (2008) describe eight dimensions of implementation: (1) Fidelity, also known as adherence or compliance, is the extent to which a program aligns with the originally intended curriculum; (2) Dosage is the amount of the original program that was provided, often measured by the number of program sessions delivered; (3) Quality is how well, clearly, and correctly the program was delivered; (4) Participant responsiveness is the extent to which the program stimulates interest and garners the attention of the participants; (5) Program differentiation refers to the uniqueness of the program from other interventions; (6) Monitoring of comparison conditions is the documentation of the services received by those outside of the intervention group; with researchers primarily focused on the intervention condition, the control group often goes unmonitored, yet knowing the activities of both groups is important when drawing conclusions about the comparative effect of a program; (7) Program reach refers to the proportion of involvement of individuals in a population and the representativeness of program participants, which is particularly important when considering program scale-up; (8) Adaptation refers to the changes made to the program that result in differences between that implementation and the original intervention. Most school-based implementation research has focused on the first two dimensions – fidelity and dosage – little is known about the effects of these other dimensions on program outcomes (Gould et al., 2016). The present study expands the field of implementation science by examining whether teachers’ occupational health and perceptions of program feasibility influence both the dosage and quality of implementation of the PAX GBG and MTP.

### 1.4. Predictors of implementation

Domitrovich et al. (2008) offered a multi-level conceptual framework outlining factors influencing the implementation of school-based interventions to guide implementation research in this context. Informed by ecological systems models (e.g., Bronfenbrenner, 1994), the Domitrovich et al. conceptual model posits that the implementation of school-based programs is impacted by influences specific to the context in which the program is being implemented. These influences are described in three main categories: individual-level factors relating to the program implementer (e.g., occupational health, perceptions of the program), school-level factors (e.g., school culture, resources), and macro-level factors (e.g., federal, state, and district policies). As most school-based intervention programs are implemented by classroom teachers instead of an external provider such as a clinician or school counselor (Forman et al., 2009), it is important to understand the role that teachers play in impacting implementation. As such, the present study focused on how the characteristics of the teachers implementing the PAX GBG + MTP may impact their dosage and quality of implementation of the programs. We focus on teachers’ occupational health and their perceptions of the feasibility of the intervention due to their theoretical and empirical relevance to teachers’ capacity to implement intervention programming, described in detail below.

#### 1.4.1. Teachers’ occupational health

Teachers’ occupational health refers to their evaluations of various aspects of their job (van Horn et al., 2004). The multifaceted construct incorporates affective, cognitive, professional, social, and psychosomatic dimensions (van Horn et al., 2004). Notably, Kazdin (1993) posited that the absence of dysfunction does not reflect the presence of optimal functioning. Thus, it is important to consider both negative experiences of distress (e.g., stress) and positive experiences of well-being (e.g., perceived ability) in assessing occupational health. Indeed, researchers have used a variety of measures to assess this construct, including job stress, burnout, self-efficacy, and others (van Horn et al., 2004; Bakker and Rodríguez-Muñoz, 2010). Although research often considers teachers’ occupational health as an outcome of an intervention (e.g., Ross et al., 2012) teachers’ occupational health may also influence their classroom practice. Specifically, the conceptual model of the Prosocial Classroom (Jennings and Greenberg, 2009) posits that teachers’ occupational health and well-being likely support effective implementation of intervention programs, while feelings of stress and associated experiences jeopardize implementation. We focus on three salient experiences of occupational health in the present study: teachers’ experiences of job stress, burnout, and perceptions of teaching ability.

##### 1.4.1.1. Job stress

Teachers report one of the highest levels of stress of any profession (Johnson et al., 2005). The Jobs Demands-Resource model of occupational stress posits that when the demands of a job exceed the resources of the individual and organization, stress can occur (Bakker and Demerouti, 2007). Theoretically, when teachers are under stress their emotional resources, attention, and cognitive energy are devoted to coping, leaving fewer resources for maintaining healthy relationships with students, supporting student development, and effectively implementing programs (Boekaerts, 1993; Roeser et al., 2012, 2021). Although empirical studies investigating these associations are still emerging, initial evidence provides support for this theory. Stressed teachers report more barriers to implementing evidence-based programs, such as lack of time to implement the program than their less-stressed counterparts (McGoey et al., 2014). Further, another study found a well-being intervention for teachers reduced stress and also improved their implementation of an evidence-based program for their students (Larson et al., 2018). Finally, one recent study found high levels of teacher stress to be associated with poor implementation quality of a mindfulness curriculum for students (Braun et al., 2023). However, this association was attenuated when teachers were provided with in-depth training, suggesting that highly stressed teachers may need more hands-on support than is typical to implement interventions well.

##### 1.4.1.2. Burnout

Prolonged exposure to stressors (e.g., job demands exceeding resources) are often associated with experiences of burnout (Maslach and Jackson, 1981). Burnout is often characterized by three dimensions: emotional exhaustion, depersonalization (e.g., feeling disconnected), and personal accomplishment (e.g., feelings of competence in the classroom; Maslach and Jackson, 1981). Burnout is an unfortunately common experience for teachers (García-Carmona et al., 2019; Salmela-Aro et al., 2019). Teachers’ feelings of burnout have been known to be associated with other salient experiences for themselves (e.g., depression, job dissatisfaction, disengagement; Leiter and Durup, 1994; Bakker and Schaufeli, 2000; Pines and Keinan, 2005), their classroom practices (e.g., poor student-teacher interactions, classroom management characterized by harsh discipline; Reinke et al., 2013) and student outcomes (e.g., impaired academic achievement; Chang and Davis, 2009; Breeman et al., 2015; Herman et al., 2020).

One process through which burnout may have such effects on students is by reducing their capacity to effectively implement evidence-based intervention programs, as posited by the Prosocial Classroom model (Jennings and Greenberg, 2009). Indeed, research from the field of social and emotional learning has supported this conceptual model; teachers’ feelings of burnout have been related to lower dosage implementation of several different school-based programs, including the PAX GBG (Ransford et al., 2009; Domitrovich et al., 2015; Swift et al., 2017). Although one study found no main effect of burnout, the effect of burnout on implementation dosage was moderated by teacher-coach alliance: burnout was associated with a lower dosage of the PAX GBG, specifically when teacher-coach alliance was low (Wehby et al., 2012). Notably, the negative effect of burnout was found in the PAX GBG dosage but not the quality of implementation (Domitrovich et al., 2015). The present study is the first to assess the role of burnout in the implementation of the combined PAX GBG + MTP interventions.

##### 1.4.1.3. Perceived ability

Self-efficacy refers to teachers’ belief in their capability to successfully accomplish a specific teaching task (Tschannen-Moran et al., 1998). Two primary domains comprise self-efficacy: (1) self-perception of teaching competence (i.e., a teacher’s assessment of their own skills and knowledge), and (2) beliefs about the demands of a specific teaching task (e.g., a teacher’s context-specific assessment of external resources and barriers). Teachers’ sense of self-efficacy is associated with other indices of occupational health (e.g., burnout, job satisfaction; Brouwers and Tomic, 2000; Skaalvik and Skaalvik, 2010, 2014). In addition, teachers’ self-efficacy is associated with the use of more supportive classroom management practices, higher quality interactions with students, and student achievement (Swars et al., 2006; Zee and Koomen, 2016). Of focus in the present study is teachers’ perceived teaching ability. Perceived teaching ability captures the teachers’ views toward their own abilities as a teacher, which may be likened to the self-perception of teaching competence domain of self-efficacy.

Although no research has investigated the association between teachers’ perceived ability and intervention implementation specifically, previous studies have explored the association between overall self-efficacy and implementation of school-based interventions. Teachers’ self-efficacy has been associated with both the quality (Rohrbach et al., 1993; Kallestad and Olweus, 2003) and dosage (Ransford et al., 2009; Clayback et al., 2022) of school-based interventions. Some studies have focused specifically on the association between self-efficacy for classroom management and implementation, with mixed results. One study of early childhood teachers’ implementation of a SEL program found self-efficacy for classroom management predictive of dosage, but not the quality of implementation (Thierry et al., 2022). Perhaps most relevant to the present investigation is a study of the PAX GBG, which found self-efficacy for classroom management was unrelated to implementation dosage and quality (Domitrovich et al., 2015). Further research is needed to clarify whether distinct aspects of self-efficacy, such as perceived teaching ability (vs. efficacy for classroom management, etc.), are associated with implementation dosage and quality.

#### 1.4.2. Perceptions of program feasibility

Perceptions of the feasibility of a program are a core component of the social validity of an intervention. Social validity refers to the extent to which an intervention is useable, valuable, and favorably viewed by interested parties (Kazdin, 1977; Wolf, 1978; Horner et al., 2005). Although teachers’ ratings of the feasibility of a specific program may be averaged to reflect the feasibility of the program as a whole, teachers themselves may vary in the extent to which they personally find the program to be feasible to implement (Han and Weiss, 2005). This teacher-level variation in perceptions of feasibility has important implications for implementation: Conceptual understandings and empirical evidence indicate that teachers who have positive perceptions of an intervention attend more training sessions (dosage) and implement the program with higher fidelity (Han and Weiss, 2005; Clayback et al., 2022). With regard to the social validity of PAX GBG specifically, previous research has found that teachers who perceive the program more favorably implement the program with greater fidelity and quality (Wehby et al., 2012). The predictive utility of social validity in the context of the combined PAX GBG + MTP has not yet been assessed and is important to consider as the combined high-quality implementation of these programs could be profound. Further, as social validity is in response to the program itself, these perceptions are potentially malleable, and results could inform amendments to the program to maximize social validity if found to be an important predictor of implementation. To this end, the present study focused on teachers’ perceptions of the feasibility of the combined PAX GBG + MTP program (i.e., how easy it was to use).

#### 1.4.3. The potential moderating effect of perceptions of feasibility on the association between stress and implementation

Although stress is theorized to be a barrier to implementation, it is possible that the effects of these predictors of implementation are more complex; a selection of factors may work together to impact implementation (Jennings and Greenberg, 2009; Ransford et al., 2009). For example, Dreer et al. (2017) found teachers’ perceptions of the program to moderate the association between teachers’ readiness to engage in the program and their commitment to utilizing new skills, where teachers experienced the greatest commitment to utilizing new skills when they were both ready to engage with the program and had positive perceptions of the program. In the context of the present study, teachers’ positive perceptions of the feasibility of the programs could serve to buffer against the negative effect of stress on implementation. Conceptually, teachers’ perceptions of feasibility may motivate teachers’ engagement with the program (Wehby et al., 2012), despite their stress and function as a protective factor to lessen the impact of stress on implementation. In contrast, high levels of stress and perceptions that the program is difficult to implement may indicate compounding risks for poor implementation. Yet, most research examining the predictors of teachers’ implementation has focused on the main effects (e.g., Domitrovich et al., 2015). Although few studies have investigated these more complex associations, one such study did find that teachers with high levels of burnout and negative perceptions of a social and emotional learning program exhibited the lowest implementation dosage and quality (Ransford et al., 2009), suggesting that these associations may be more complex than initially proposed (Jennings and Greenberg, 2009). Thus, additional research probing these more complex effects will contribute to our understanding of how combinations of factors may work together to impact implementation. Although we may hypothesize that perceptions of feasibility may buffer against the negative effects of burnout in the same way as it might for stress, as burnout emerges from experiences of chronic stress (Maslach and Jackson, 1981), stress, rather than burnout, is likely a more salient experience for early career teachers. Thus, of interest in this study was whether early career teachers’ perceptions of feasibility may attenuate the negative effects of stress on implementation.

### 1.5. Present study

The present study aimed to expand our understanding of the role that teachers play in the implementation of evidence-based programs for youth by investigating predictors of implementation. Guided by the Domitrovich et al. (2015) conceptual model of implementation of school-based interventions and the Prosocial Classroom model (Jennings and Greenberg, 2009), the present study explored the association between teachers’ occupational health and perceptions of feasibility in relation to their dosage and quality of implementation of the PAX GBG + MTP program. Specifically, we addressed the following two specific research questions: RQ_1_ Do teachers’ own occupational health and perceptions of the feasibility (i.e., ease of use) of the program impact their implementation of the PAX GBG + MTP? RQ_2_) Is the effect of teachers’ stress on their implementation of the PAX GBG + MTP moderated by their perceptions of the feasibility of the program? We hypothesized that low levels of job stress, low levels of burnout, high levels of perceived ability, and positive perceptions of feasibility would be associated with greater dosage and implementation quality. We also hypothesized that the negative association between stress and implementation would be weaker for teachers who had positive perceptions of feasibility.

## 2. Methods

### 2.1. Study design and recruitment

The present study draws from a longitudinal, teacher-level randomized controlled trial of the PAX GBG + MTP program. Early career teachers (≤ 3 years of teaching experience) hired by three participating public school districts in Kindergarten-3^rd^ grade were identified by the districts. These teachers were recruited into the project by project staff during district-wide professional development events for early-career teachers held prior to the start of the 2013 school year. To limit heterogeneity in teaching demands, eligible teachers included those in early grades (Kindergarten-3^rd^ Grade) and excluded Teach for America engaged teachers, given the variation in their educational backgrounds from typical teachers. Project staff conducted all recruitment sessions, either through attendance at new teacher training and orientation events or through individual or small group sessions. Participation was voluntary, and teachers provided written informed consent consistent with IRB procedures approved at the investigators’ universities and school divisions. Participating teachers received an honorarium (e.g., gift cards) for their participation and completion of data collection activities. This recruitment and randomization procedure was repeated the following 2 years (i.e., the fall of 2014 and 2015, respectively), for a total of three cohorts.

Recruitment efforts resulted in 272 interested teachers, of which 236 teachers consented to participate. Of those, eight withdrew before randomization, 15 were ineligible due to being assigned to an ineligible classroom (i.e., not Kindergarten-3^rd^ Grade, special education classroom, resource class), not being permitted to attend training (based on the principal’s decision), having already been trained in the PAX GBG or MTP, leaving the participating districts, or leaving the teaching profession altogether. Of those eligible, 25 left the project prior to baseline data collection. The final sample in the RCT intent-to-treat analyses included 188 teachers (69% of initially interested teachers; 80% of those who consented) recruited from 72 schools (Median number of teachers per school = 2, Range = 1–13 teachers). Cohort 1 consisted of 56 teachers (30 control condition, 26 intervention condition) from 34 schools, Cohort 2 consisted of 51 (25 control condition, 26 intervention condition) teachers from 30 schools, and Cohort 3 consisted of 81 teachers (39 control condition, 42 intervention condition) from 36 schools. Note that the same school could be represented in multiple cohorts if they had new teachers in subsequent years, as was the case in several instances. See Tolan et al. (2020) for the full consort diagram. Attrition during the first study year was low, with 11% (10 from control, 11 from intervention) discontinuing participation before the Year 1 post-intervention timepoint.

### 2.2. Participants

Due to the present study’s focus on implementation outcomes, only the 94 teachers randomized to the intervention condition were included in the analytic sample in this study. Randomization was effective as there were no significant differences in baseline demographics, occupational health, nor implementation outcomes between teachers in the intervention and control conditions (see Downer et al., 2023). The majority of teachers in the intervention condition were female (93%) and White (80%), with 1–3 years of teaching experience, and most teachers were in their first year of teaching (60%). Teachers were approximately evenly distributed across the Kindergarten-3^rd^ Grade classes.

### 2.3. Procedure

Baseline (Time 1) data collection occurred in the fall of the school year as close to the beginning of school as possible, in October. Post-intervention (Time 2) data collection occurred 7 months later, in May, shortly before the end of the school year. At each timepoint, teachers completed an online survey, and trained observers conducted classroom observations. Following baseline data collection, teachers were randomly assigned (blocking on school and district) to the intervention or control conditions. School-level demographic data were obtained from the state department of education.

#### 2.3.1. Classroom observation procedures

Observations were conducted in accordance with the protocol for the Classroom Assessment Scoring System (CLASS; Pianta et al., 2008a). At each timepoint, certified CLASS observers conducted 4–6, 15-min observation cycles. Observations were conducted over two separate days balancing observations in the morning and the afternoon. Immediately following each observation cycle, observers stepped out of the classroom and completed the CLASS ratings. There were no calculations of inter-rater agreement following the CLASS training and certification process, but previous research has demonstrated high inter-rater agreement for the CLASS (79–94% within 1 point; Pianta et al., 2008b; Mantzicopoulos et al., 2018).

#### 2.3.2. PAX GBG

The GBG allows teachers to utilize social learning principles within a team-based, game-like context to reduce aggressive, disruptive, and off-task behavior and facilitate academic instruction. The current project used the PAX GBG, an augmented version of GBG which integrates ancillary components known to improve compliance and classroom management (Embry, 2002).

Prior to the implementation of the PAX GBG at the beginning of the school year, the teachers and students collaborated to define their vision of a “PAX” (Latin, meaning peaceful or ideal) classroom. Toward that end, they identified the behaviors that were necessary for creating a focused, productive, and peaceful classroom. During this collaboration, the teacher explained to the students that the positive behaviors they listed were referred to as “PAX” behaviors, and the negative behaviors were referred to as “spleems.” After jointly defining PAX and spleems, teachers assigned students to one of three or four teams. The teams worked cooperatively to maintain PAX behavior in the classroom. Teachers gave points to the team when a member displayed a spleem. Teachers were trained to respond unemotionally to rule-breaking and when marking points against a child’s team. At the end of the game period, all teams with three or fewer spleems won the game. The students were rewarded for displaying self-control, emotion regulation, and group regulation while not attending to or reinforcing the misbehavior of others. The team-based nature of the game allowed teachers to take advantage of positive peer pressure to improve academic and pro-social student behavior at the individual as well as at the classroom level.

##### 2.3.2.1. Training in the PAX GBG

Professional development was provided to the intervention teachers over the course of one weekend day, during which teachers received intensive training and practice using the PAX GBG approach aligned with the MTP framework. Seventy-seven percent of the intervention teachers completed this training, with the remainder attending a small group or one-on-one make-up trainings. Teachers were asked to play approximately three PAX GBG games each school day with increasing length and in increasingly varied settings over the course of the year.

#### 2.3.3. MTP

MTP is grounded in an evidence-based framework for thinking about teacher-student interactions that contribute to student behavior and achievement, called Teaching through Interactions (Hamre et al., 2013). This framework emphasizes that interactions should be emotionally supportive, well-organized, and cognitively enriching. The Teaching through Interactions framework is based on these three core domains of classroom interactions as captured by an observational approach called the Classroom Assessment Scoring System (CLASS): emotional support, instructional support, and classroom organization (Pianta et al., 2008a). During the intervention, the CLASS is used as a lens for viewing and providing feedback on a teacher’s practice in the classroom.

##### 2.3.3.1. Training in MTP

Teachers in the intervention condition also participated in 1 day of training in MTP. Following the training, teachers participated in biweekly MTP coaching cycles throughout the training year, with initial contact in-person that then shifted to web-mediated training. See Tolan et al. (2020) for a detailed description of coaching steps. Over the course of the school year, these coaching cycles focused on all three CLASS domains as well as elements of the PAX GBG that would help teachers optimize their implementation of the PAX GBG by attending to their interactions with students. The coaching cycles were intended to be collaborative, supportive, constructive, and to help teachers develop CLASS and PAX GBG knowledge, improve observation skills, develop analysis skills, feel supported in these endeavors, and increase their sense of agency and efficacy in the classroom. Teachers were asked to incorporate new strategies related to both the CLASS domains and PAX GBG elements into their teaching practice to improve both their implementation of the PAX GBG and their overall teaching practice.

### 2.4. Measures

#### 2.4.1. Implementation outcomes

The present study reports implementation dosage and quality data for the PAX GBG and MTP collected at post-intervention.

##### 2.4.1.1. Implementation dosage

The number of *coaching cycles completed* is an indicator of the dosage of MTP teachers received. These data were collected on each participating teacher through the online MTP coaching platform. The number of *PAX GBG games played* is an indicator of the dosage of the PAX GBG the students received. For the school year following the training, teachers self-reported on the number of PAX GBG games played each week, which was averaged across the school year.

##### 2.4.1.2. Implementation quality

Following the CLASS procedure above, after each classroom observation period, observers provided a rating from 1 to 7 (1 = Low, 7 = High) for each of the 11 CLASS dimensions. In accordance with contemporary uses of the CLASS (e.g., Mashburn et al., 2008), three scales were created to reflect the core CLASS domains: classroom management, emotional support, and instructional support. Teachers’ scores within each timepoint (baseline, post-intervention) were averaged across cycles of observation. Cronbach’s α for all domains and timepoints were acceptable (ranging from 0.84–0.92). As MTP aims to improve the quality of teachers’ interactions with students using the CLASS as a guide to anchor coaches’ feedback to teachers, the CLASS was used as an indicator of the quality of teachers’ implementation of MTP.

###### 2.4.1.2.1. Emotional support

Emotional Support was calculated as the average of the positive climate, negative climate, teacher sensitivity, and regard for student perspectives dimensions.

###### 2.4.1.2.2. Instructional support

Instructional support reflects teachers’ facilitation of academic learning, measured as the average of the quality of feedback, concept development, and language modeling.

###### 2.4.1.2.3. Classroom organization

Classroom organization, also referred to as classroom management, assesses the quality of teachers’ interactions with students while they are managing the students in the room. It is the average of the behavior management, productivity, and instructional learning formats dimensions.

#### 2.4.2. Occupational health and perceptions of feasibility

Main predictors of interest included indices of teachers’ occupational health collected at baseline, namely job stress, burnout, and perceived ability, along with teachers’ perceptions of intervention feasibility collected post-intervention.

##### 2.4.2.1. Job stress

Teachers’ experiences of job stress were assessed using items from the National Institute for Occupational Safety and Health survey of work-related stress (National Institute for Occupational Safety and Health, 1999). Teachers rated five items about their current feelings of stress (e.g., “In my job, I feel like I am under great stress”) on a 1 to 4 scale (1 = Strongly Disagree; 4 = Strongly Agree; α = 0.82).

##### 2.4.2.2. Burnout

Teachers’ experiences of burnout were assessed using four items from the emotional exhaustion subscale of the Maslach Burnout Inventory (Maslach et al., 1996). Teachers rated four items about their feelings of emotional exhaustion (“I feel burned out from my work,” “I feel like I am at the end of my rope,” I feel emotionally drained from my work,” and “I feel used up at the end of the work day”) on a 1 to 4 scale (1 = Strongly Disagree; 4 = Strongly Agree; α = 0.85). This subscale was abbreviated for use in this study due to practical considerations to reduce participant burden.

##### 2.4.2.3. Perceived ability

Teachers’ perceptions of their ability as a teacher were assessed using the Perceived Ability subscale of the Factors Influencing Teaching Choice (FIT-Choice) measure (Watt and Richardson, 2007). Teachers rated three items about their perceived ability (“I have the qualities of a good teacher,” “I have good teaching skills,” and “Teaching is a career suited to my abilities”) on a 1 to 4 scale (1 = Strongly Disagree; 4 = Strongly Agree; α = 0.69).

##### 2.4.2.4. Perceptions of feasibility

Perceptions of the feasibility of the combined PAX GBG + MTP intervention were assessed using items from the Teacher Perceptions of the Intervention Attributes scale (Domitrovich et al., 2015) with adapted wording to be relevant to the PAX GBG and MTP programs. Teachers rated five items assessing their perceptions of how feasible the combined program was to implement (e.g., “The GBG + MTP coaching process was easy to participate in”) on a 1 to 4 scale (1 = Strongly Disagree; 4 = Strongly Agree; α = 0.72).

#### 2.4.3. Demographics

At the teacher-level, teachers self-reported the grade that they taught and their years of teaching experience. School-level demographic data regarding the enrollment of the school and percent of students eligible for free and reduced-priced meals (FARMS) were obtained from the state department of education.

### 2.5. Analytic plan

#### 2.5.1. Missing data

Full information maximum likelihood (FIML) was used to incorporate all participants with baseline data (including those who did not provide data at post-intervention) into the intent-to-treat analyses. This approach accounts for the missing data while obtaining minimally biased estimates (e.g., Little et al., 2014; Witkiewitz et al., 2014).

#### 2.5.2. Preliminary analyses

All analyses were run in R studio. Preliminary analyses included descriptive statistics and bivariate correlations among study measures.

#### 2.5.3. RQ1: The association between occupational health, perceptions of feasibility, and implementation

Multiple linear regression models with cluster robust standard errors were employed to test the association between teachers’ occupational health (i.e., stress, burnout, perceived ability) and perceptions of feasibility and their implementation of the PAX GBG + MTP. Because FIML was invoked to account for missing data, models were run in the latent framework using the lavaan package (Rosseel, 2012). A separate model was run for each outcome. Cluster robust standard errors were used to account for the nesting of teachers in schools in these analyses, where multilevel modeling is not appropriate given the average cluster size was so small (*M* = 2.44 teachers/school). Models predicting the number of coaching cycles completed used estimator = “MLR” to account for the non-normal distribution of this count outcome. Measures of occupational health and perceptions of feasibility were grand mean centered. Models controlled for grade level (continuous, where 0 = Kindergarten) and years teaching (continuous, where 0 = 1^st^ year), as the participants in this study ranged from Kindergarten-3^rd^ Grade teachers and were in their 1^st^-3^rd^ years of teaching. Models predicting implementation quality (CLASS outcomes) controlled for teachers’ CLASS scores at baseline, which were grand mean centered. School-level covariates included the school enrollment and the percent of students eligible for FARMs, which were standardized, and grand mean centered, respectively. Centering in this way results in an intercept that can be interpreted as the predicted level of implementation for a teacher who is experiencing an average level of job stress, burnout, perceptions of their teaching ability, and perceptions of feasibility, and is a Kindergarten teacher in their 1st year in the classroom, who is in a school of average enrollment and eligibility for FARMs.

#### 2.5.4. RQ2: The moderating effect of perceptions of feasibility on the association between stress and implementation

To test the potentially moderating role of perceptions of feasibility, the interaction of stress and perceptions of feasibility was added to each of the models above.

## 3. Results

### 3.1. Preliminary analyses

Descriptive statistics of measures are provided in Table 1. Teachers demonstrated a relatively high dosage of the MTP elements of the combined program. On average, teachers completed 8.24 coaching cycles, which exceeded the target number of cycles of 8, which previous research has shown to impact teacher practice and student outcomes (Allen et al., 2011, 2015). Implementation of the PAX GBG elements of the combined program was also relatively high, with teachers playing an average of 9 games per week (i.e., an indicator of dosage).

**Table 1:** Descriptive statistics.

	*N*	*Missing*	Mean	*SD*	Minimum	Maximum	Range
Implementation outcomes (Time 2)							
Implementation dosage							
Coaching cycles completed	86	8%	8.24	2.3	1	11	10
Number of games played	83	11%	9.16	2.81	2.11	15.43	13.32
Quality of implementation							
Emotional support	83	11%	4.88	0.68	2.39	6.25	3.86
Classroom organization	83	11%	5.04	0.71	3.38	6.28	2.90
Instructional support	83	11%	2.35	0.65	1.33	4.22	2.89
Occupational health (Time 1)							
Job stress	91	3%	2.63	0.61	1.00	3.80	2.80
Burnout	91	3%	2.71	0.69	1.00	4.00	3.00
Perceived ability	91	3%	3.34	0.44	2.00	4.00	2.00
Feasibility (Time 2)							
Easy to use	78	16%	3.16	0.56	1.60	4.00	2.40

Bivariate correlations among study measures are provided in Table 2. Notably, burnout was significantly negatively correlated with the number of MTP coaching cycles completed (*r* = −0.21, *p* = 0.048), whereas perceptions of feasibility were significantly positively correlated with the number of MTP coaching cycles completed (*r* = 0.36, *p* = 0.001). These correlations indicated that teachers who reported higher levels of burnout at baseline completed fewer coaching cycles than their peers who were more burned out, and that teachers who reported the program was more feasible to implement (i.e., easy to use) completed more coaching cycles than their peers who reported lower levels of program feasibility.

**Table 2:** Bivariate correlations among study measures.

		Implementation outcomes					Occupational health			Feasibility	Demographics			
		Implementation dosage		Quality of implementation							Teacher level		School level	
		1	2	3	4	5	6	7	8	9	10	11	12	13
Implementation outcomes (Time 2)														
Implementation dosage														
1	Coaching Cycles Completed													
2	Games Played	**0.26**												
Quality of implementation														
3	Emotional support	0.19	−0.09											
4	Classroom organization	0.08	0.03	**0.52**										
5	Instructional support	0.05	−0.11	**0.75**	**0.46**									
Occupational health (Time 1)														
6	Job Stress	−0.17	−0.02	0.12	0.13	0.00								
7	Burnout	**−0.21**	0.00	0.06	0.17	−0.08	**0.84**							
8	Perceived Ability	0.10	0.03	−0.04	−0.06	−0.06	**−0.25**	**−0.29**						
Feasibility (Time 2)														
9	Feasibility	**0.36**	−0.02	−0.01	0.00	−0.06	0.05	−0.05	0.08					
Demographics														
Teacher level														
10	Grade	−0.15	−0.05	0.09	0.16	0.14	0.17	0.19	−0.12	0.20				
11	Years teaching	−0.16	0.05	0.13	0.01	0.03	0.19	0.15	−0.04	−0.12	−0.09			
School level														
12	Enrollment	−0.02	−0.10	0.13	−0.07	−0.10	−0.01	0.05	0.08	−0.16	0.04	0.09		
13	Eligible for FARMS	−0.10	**0.35**	**−0.25**	−0.14	**−0.31**	0.08	0.05	0.18	−0.13	**−0.21**	0.12	−0.15	

Bold indicates significant at *p* < 0.05. FARMS = Free and Reduced-Priced Meals.

### 3.2. RQ1: The association between occupational health, perceptions of feasibility, and implementation

#### 3.2.1. Coaching cycles completed

Teachers’ perceptions of the feasibility of the program were associated with attending more coaching cycles (*B* = 1.66, *SE* = 0.53, *p* = 0.002). Grade was negatively associated with the number of coaching cycles completed (*B* = −0.43*, SE* = 0.19, *p* = 0.03; Table 3), such that teachers in lower grades attended more coaching sessions. No other effects were significant.

**Table 3:** Main effects models: predicting implementation dosage and quality of PAX GBG + MTP.

	Implementation dosage				Implementation quality					
	Coaching cycles completed		Number of games played		Emotional support		Instructional support		Classroom organization	
	*B*	*SE*	*B*	*SE*	*B*	*SE*	*B*	*SE*	*B*	*SE*
Intercept	9.10*	0.41	9.13*	0.50	4.74*	0.12	2.31*	0.15	4.89*	0.11
Occupational health										
Job stress	−0.08	0.53	−0.38	0.86	0.13	0.21	0.07	0.28	0.18	0.22
Burnout	−0.35	0.55	0.16	0.87	0.00	0.17	0.16	0.22	−0.14	0.16
Perceived ability	−0.01	0.61	−0.29	0.70	0.09	0.19	0.04	0.15	−0.03	0.12
Feasibility										
Feasibility	1.66*	0.53	0.11	0.51	−0.07	0.13	−0.07	0.13	−0.20	0.15
Demographics and covariates										
Teacher level										
Grade	−0.43*	0.19	0.07	0.31	0.07	0.05	0.04	0.07	0.11^+^	0.06
Years teaching	−0.29	0.32	−0.08	0.34	0.09	0.08	0.00	0.09	0.00	0.09
CLASS (where appropriate)					0.40*	0.12	0.40*	0.12	0.34*	0.09
School level										
Enrollment	0.12	0.20	−0.16	0.34	0.04	0.06	−0.09	0.07	−0.11*	0.05
FARMS	−0.44	1.01	5.39*	1.47	−0.66^+^	0.39	−0.27	0.32	−0.74*	0.34
R Squared	0.24		0.15		0.29		0.17		0.30	

* indicates significant at *p* < 0.05, + indicates significant at *p* < 0.10. Cohort was omitted in final models because its inclusion did not substantively change the pattern of results. FARMS = Free and Reduced-Priced Meals.

#### 3.2.2. Number of games played

Occupational health and perceptions of feasibility were unrelated to the number of PAX GBG games played. At the school level, the percent of students eligible for FARMS was associated with playing more games (*B* = 5.39, *SE* = 1.47, *p* < 0.001). No other effects were significant.

#### 3.2.3. Emotional support

Occupational health and perceptions of feasibility were unrelated to observations of teachers’ emotional support. Teachers’ emotional support at baseline was strongly associated with their emotional support at post-intervention (*B* = 0.40, *SE* = 0.12, *p* < 0.001). At the school level, the percent of students eligible for FARMS was marginally negatively associated with emotional support (*B* = −0.66, *SE* = 0.39, *p* = 0.09). No other effects were significant.

#### 3.2.4. Instructional support

Teachers’ instructional support at baseline was strongly associated with their emotional support at post-intervention (*B* = 0.40, *SE* = 0.12, *p* < 0.001). No other effects were significant.

#### 3.2.5. Classroom organization

Occupational health and perceptions of feasibility were unrelated to observations of teachers’ classroom organization. Grade level was marginally associated with classroom organization (*B* = 0.34, *SE* = 0.09, *p* < 0.001) such that teachers in higher grades were observed to have higher levels of classroom organization. Teachers’ classroom organization at baseline was strongly associated with their classroom organization at post-intervention (*B* = 0.34, *SE* = 0.09, *p* < 0.001). At the school level, school enrollment (*B* = −0.11*, SE* = 0.05*, p* = 0.03) and the percent of students eligible for FARMS (*B* = −0.74, *SE* = 0.34, *p* = 0.03) were negatively associated with classroom organization such that the larger the school and the more students eligible for FARMS, the lower the observed classroom organization. No other effects were significant.

### 3.3. RQ2: The moderating effect of perceptions of feasibility on the association between stress and implementation

#### 3.3.1. Coaching cycles completed

The interaction between stress and feasibility was not significant in predicting the number of coaching cycles completed (*B* = 0.17, *SE* = 1.04, *p* = 0.87; Table 4).

**Table 4:** Moderation models: predicting implementation dosage and quality of PAX GBG + MTP.

	Implementation dosage				Implementation quality					
	Coaching cycles completed		Number of games played		Emotional support		Instructional support		Classroom organization	
	*B*	*SE*	*B*	*SE*	*B*	*SE*	*B*	*SE*	*B*	*SE*
Intercept	9.12*	0.40	9.16*	0.50	4.72*	0.12	2.28*	0.15	4.86*	0.12
Occupational health										
Job stress	−0.05	0.53	−0.32	0.89	0.09	0.21	−0.01	0.27	0.13	0.22
Burnout	−0.35	0.56	0.14	0.88	0.02	0.17	0.18	0.22	−0.13	0.16
Perceived ability	−0.01	0.61	−0.28	0.70	0.08	0.19	0.03	0.16	−0.05	0.12
Feasibility										
Feasibility	1.64*	0.51	0.06	0.53	−0.04	0.12	0.00	0.13	−0.16	0.15
Interactions										
Stress*Feasibility	0.17	1.04	0.51	0.86	−0.33	0.27	−0.77*	0.26	−0.41^+^	0.24
Demographics and covariates										
Teacher level										
Grade	−0.44*	0.18	0.05	0.31	0.08	0.06	0.05	0.07	0.12*	0.06
Years teaching	−0.30	0.31	−0.11	0.34	0.11	0.08	0.03	0.09	0.02	0.09
CLASS (where appropriate)					0.40*	0.11	0.48*	0.11	0.35*	0.09
School level										
Enrollment	0.12	0.20	−0.16	0.35	0.03	0.06	−0.11^+^	0.07	−0.11*	0.05
FARMS	−0.47	1.02	5.32*	1.43	−0.61	0.40	−0.10	0.34	−0.67^+^	0.36
R Squared	0.24		0.15		0.30		0.26		0.33	

* indicates significant at *p* < 0.05, + indicates significant at *p* < 0.10. Cohort was omitted in final models because its inclusion did not substantively change the pattern of results. FARMS = Free and Reduced-Priced Meals.

#### 3.3.2. Number of games played

The interaction between stress and feasibility was not significant in predicting the average number of PAX GBG games played each week (*B* = 0.51, *SE* = 0.86, *p* = 0.55).

#### 3.3.3. Emotional support

The interaction between stress and feasibility was not significant in predicting observations of teachers’ emotional support (*B* = −0.33, *SE* = 0.27, *p* = 0.21).

#### 3.3.4. Instructional support

**Figure 1:**
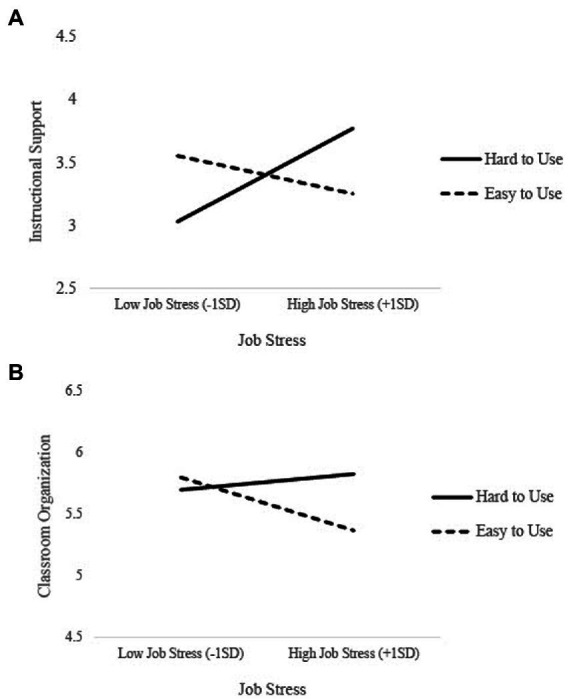
The effect of job stress on implementation quality was moderated by teachers’ perception of feasibility of PAX GBG + MTP. Teachers experiencing high levels of stress and felt the program was harder to use demonstrated greater **(A)** instructional support, and **(B)** classroom organization than those who were highly stressed and reported the program was easy to use.

The interaction between stress and feasibility was significant in predicting observations of teachers’ instructional support (*B* = −0.77, *SE* = 0.26, *p* = 0.003). This effect, visualized in Figure 1A, indicates that teachers who reported high levels of stress and lower levels of program feasibility (i.e., perceptions that the program was harder to use) implemented the program with higher quality than those who were highly stressed and reported the program was more feasible to implement (i.e., easy to use).

#### 3.3.5. Classroom organization

The interaction between stress and feasibility was marginally significant in predicting observations of teachers’ classroom organization (*B* = −0.41, *SE* = 0.24, *p* = 0.09). This effect, visualized in Figure 1B, shows that although job stress was relatively unrelated to implementation quality for those teachers who reported lower levels of program feasibility (i.e., perceptions that the program was harder to use), the opposite was true for teachers who reported that the interventions were feasible to implement. That is, teachers who had greater perceptions of program feasibility (i.e., perceptions that the program was easy to use) and experienced higher stress had poorer quality implementation than those who had greater perceptions of program feasibility and experienced low stress.

## 4. Discussion

The present study investigated the associations among teachers’ occupational health and perceptions of the feasibility of the combined PAX GBG + MTP program and two dimensions of implementation: dosage and quality. Contrary to our hypotheses, there were no main effects of any occupational health indicator on implementation dosage or quality. However, teachers who reported that the program elements were easier to use did complete more coaching cycles. In addition, the effect of teachers’ job stress on two dimensions of implementation quality, instructional support and classroom organization, was moderated by teachers’ perceptions of how feasible the program was, suggesting that the effect of job stress on implementation may be more nuanced than initially proposed (Jennings and Greenberg, 2009). The present study expands the field of implementation science in education research by: (1) investigating several potential predictors of implementation with a particular focus on teachers’ occupational health and perceptions of program feasibility; (2) considering two aspects of implementation: dosage and quality; and (3) testing these effects within the context of the combination of two evidence-based programs (the PAX GBG and MTP) with strong potential for scaling up and which have seldom been combined or tested together. Given that early-career teachers may have fewer resources (e.g., training, on the job experience) compared to more experienced teachers, evidence-based programs for early-career teachers may be particularly useful resources for meeting the job demands they face.

### 4.1. Teachers’ occupational health did not predict implementation

Regarding RQ_1_, although the bivariate correlations indicated that burnout at baseline was negatively associated with the number of coaching cycles completed, this association did not hold in the more complex models. It is worth noting that neither stress, burnout, nor perceived ability at baseline predicted implementation dosage or quality as assessed at post-program. Thus, the hypothesis that greater occupational health at baseline would be associated with greater implementation dosage and quality of the interventions was not supported. The lack of significant associations between occupational health and implementation found in the current study is mostly inconsistent with previous findings of school-based intervention programs, including the PAX GBG, which suggested that higher levels of occupational health were associated with greater implementation dosage and quality (Ransford et al., 2009; Wehby et al., 2012; Domitrovich et al., 2015). These previous studies have included qualitatively different populations than the current study, including teachers of up to 5^th^ grade, and those beyond their first 3 years in the classroom. Yet, results are consistent with existing studies of the PAX GBG which found no association between self-efficacy for behavior management and implementation (Domitrovich et al., 2015), and a recent study of early childhood educators which found inconsistent associations between stress and implementation (Clayback et al., 2022).

The non-significant associations between stress, burnout, perceived ability, and implementation outcomes in this study should be interpreted in the context of several key considerations. First, occupational health may yet be related to implementation, just the present study’s assessment of stress, burnout, and perceived ability may not be the relevant occupational health indicators that are important for implementation. For example, perceived ability, a specific aspect of self-efficacy measured in this study, may be too nuanced; with previous research indicating that general self-efficacy is associated with the quality and dosage of school-based interventions (Kallestad and Olweus, 2003; Ransford et al., 2009), it may be that general self-efficacy or specific self-efficacy around implementing new programs, rather than perceived teaching ability, may be related to implementation. Specifically, positive dimensions of occupational health such as job satisfaction and feelings of personal accomplishment are known to be salient experiences for teachers (e.g., Maslach et al., 2001; Hakanen et al., 2006). These indices of occupational health were not measured in the present study but may influence teachers’ implementation of intervention programs. Future research assessing positive indices of occupational health (e.g., general self-efficacy, job satisfaction, etc.) will shed light on this possibility.

Moreover, the current project was focused solely on early career teachers. Experiences of burnout may be less salient than other measures of occupational health in this population teachers given it results from experiences of chronic stress, which these teachers may not have had time to experience yet. In addition, there may be other factors besides occupational health that exert a greater influence on implementation quality for early career teachers, such as administrative support or openness to interventions. Future research should explore other such teacher-specific factors that may influence implementation quality among early career teachers (Domitrovich et al., 2008). Further, because these teachers are still actively developing their teaching practices, efforts to optimize implementation during this period may particularly impactful. For example, interventions addressing barriers to implementation could have a positive effect on these teachers’ implementation of evidence-based programs across their career. It is also worth noting that the teachers in this study tended to report relatively high levels of occupational health. For example, teachers reported high levels of ability with limited variation (*M* = 3.34; *SD* = 0.44), potentially precluding the opportunity to detect significant differences across the spectrum of ability. Levels of burnout in this sample (*M* = 2.71; *SD* = 0.69) were also lower than those in other studies (e.g., Roeser et al., 2021), perhaps due to their early career status. Regardless, the limited variability in these measures of occupational health may have also limited their predictive utility; it may be that higher levels of stress and burnout are necessary in order to impair implementation. It is also possible that the indicators of occupational health measured in this study may have effects on other domains of implementation described by Durlak and DuPre (2008) that were not assessed in this study (e.g., participant responsiveness). Finally, the moderation of the effect of stress on implementation quality by perceptions of feasibility suggests that the association between occupational health and implementation may be more nuanced than direct effects, a finding which we explore further in the subsequent sections.

### 4.2. Teachers’ perceptions of the feasibility of the PAX GBG + MTP program predicted MTP dosage

The hypothesis that greater perceptions of program feasibility would be associated with greater implementation was partially supported. Teachers’ perceptions of how feasible the program was, an indicator of the social validity of the intervention, were not predictive of implementation quality, but were predictive of implementation dosage, assessed here as the number of coaching cycles completed. These results are consistent with existing evidence that positive perceptions of the program are associated with greater implementation (e.g., Wehby et al., 2012; Clayback et al., 2022). Implementation dosage is an important outcome to consider as existing literature has found dosage of MTP to be related to program outcomes (e.g., Pianta et al., 2014, 2022). This finding is informative for interventionists as it indicates that designing programs in ways that are simple to implement could be an effective strategy to increase sustained engagement in the program and subsequent targeted outcomes. Findings from successfully implemented school-based interventions have highlighted that school administration can be important champions for interventions (Forman et al., 2009). In this vein, it may be advantageous for school leadership to frame these programs as easy to use and easy to integrate into teaching, which could set the program up for success from the start (Forman et al., 2009).

Importantly, we measured perceptions of program feasibility alongside post-intervention assessments of implementation. This decision was made to not overburden participants with another survey in the middle of the school year. However, this design decision precludes definitive conclusions about the directionality of this association; yet, it is anticipated that teachers’ perceptions of program feasibility preceded their implementation of the games and their attendance in coaching cycles. Finally, although we conceptualized teachers’ perceptions of how easy the program elements were to use as an assessment of feasibility and social validity (Kazdin, 1977; Wolf, 1978), it could also be conceptualized as a component of implementation, namely participant responsiveness, a little studied dimension of implementation described in Durlak and DuPre (2008). The conceptualization of the construct underlies important design and analytic decisions, such as situating it as a predictor of implementation or an implementation outcome.

### 4.3. The association between stress and implementation quality was moderated by perceptions of feasibility

Regarding RQ_2_, a significant interaction between teacher-reported stress at baseline and perceptions of feasibility at post-intervention emerged in models predicting both instructional support and classroom organization, indicating that the effect of stress on implementation quality differed according to perceptions of the feasibility of the program. Although our hypothesis regarding feasibility moderating the effect of stress on implementation was supported, the direction of these effects was contrary to our hypotheses. We hypothesized that the negative association between stress and implementation would be weaker for teachers who had positive perceptions of feasibility. Yet, results indicated that highly stressed teachers demonstrated greater instructional support and classroom organization when they found the program was *harder* to use compared to teachers who found it easy to use. For instructional support, we found that teachers reporting low levels of stress had higher implementation quality when they found the program easy to use compared to low-stress teachers who found it hard to use. The findings among low-stress teachers are consistent with previous literature, which has found that positive perceptions of social validity are associated with increased implementation (Wehby et al., 2012; McNeill, 2019). Contrary to previous research, the findings among highly stressed teachers may be capturing a particular subset of highly conscientious teachers who devoted more time and effort to learning and implementing the program, thus making it more difficult to use due to the high resource burden. It may also be the case that highly stressed teachers may have perceived the program as more valuable or useful due to its perceived complexity and difficulty, thus leading these teachers to implement increased instructional support and classroom management techniques. The complexity of these findings is aligned with recent evidence that the association between teachers’ stress and the implementation of a mindfulness-based program for students was moderated by the amount of training they received (Braun et al., 2023). Together, these results suggest that there is more nuance to these associations than suggested in conceptual models, such as the Prosocial Classroom Model, in that the effect of stress on implementation may differ according to other teacher-, school-, and program-specific factors (Dreer et al., 2017; Jennings and Greenberg, 2009). Results should also be interpreted in light of the timing of these measures, which is elaborated more in the Limitations and Future Research Directions section.

### 4.4. Teacher- and school-level demographics and implementation

Although not a main focus of these analyses, the effects of teacher- and school-level demographics included as covariates yielded findings also worth discussing. Teacher-level demographics of grade level and years of teaching experience were primarily unrelated to teachers’ implementation dosage and quality. The exception was that teachers of lower grade levels completed more MTP coaching cycles. Teaching in the lower grade levels, particularly, is highly relational and high-quality interactions with students are as important as didactic instruction (Pianta and Stuhlman, 2004; Burchinal et al., 2008). Teachers of younger students could have been more motivated to attended MTP coaching because the relational content was particularly salient given the age of their students.

At the school level, teachers working in schools where more (vs. fewer) students were eligible for FARMS played a higher number of games, indicating increased implementation dosage in these schools. At the same time, implementation quality across all CLASS domains was lower for teachers in schools with more (vs. fewer) students eligible for FARMS. These results are consistent with existing research demonstrating that students experiencing the greatest socioeconomic need have teachers with lower-quality interactions (e.g., St Clair and Stone, 2016). Similarly, teachers in larger schools were observed to have lower levels of classroom organization. These findings indicate that although teachers in schools with high levels of FARMS may recognize the need for such interventions and employ more PAX GBG games than their peers from other schools, the quality of their implementation of MTP may be lower. These findings highlight that predictors of implementation dosage are not necessarily redundant with predictors of implementation quality, suggesting that researchers should continue to investigate dimensions of implementation as related yet separate outcomes. These teacher- and school-level findings could be useful in identifying teachers who may be at risk for a lower dosage of implementation and lower quality implementation of interventions.

### 4.5. Limitations and future research directions

There were certain limitations of the perceived ability measure used in the current analysis, evidenced by the relatively low internal consistency of the measure (i.e., α = 0.69). This may be due to the fact that the items were drawn from a scale intended to measure individual’s motivations for becoming a teacher, such that the items only capture the teaching competence domain of self-efficacy. Based on these findings, future research should incorporate measures that assess both domains of self-efficacy in order to capture both internal and external influences on teachers’ perceived self-efficacy. Despite this limitation, the current findings demonstrate the importance of incorporating task- and context-specific measures of self-efficacy when examining factors that influence implementation quality.

An assumption underlying the interpretations of the feasibility findings is that perceptions of feasibility were stable across the course of the intervention. As feasibility was only assessed at post-intervention in this study, we were unable to test the variability nor directionality of these effects. Perceptions of the feasibility, or more generally, the social validity, of interventions could shift over the course of the program (Clayback et al., 2022). Future research should administer measures of social validity throughout the intervention in order to understand the potentially bidirectional influence between social validity and implementation dosage and quality, and what might predict more favorable changes in social validity over the course of the intervention.

In addition, the current findings should be interpreted within the context of early career, elementary school teachers since the identified associations with implementation quality and dosage may be specific to this population. Furthermore, these findings should be contextualized within the sociodemographic makeup of the sample, given that the sample was predominantly white (80%) and female (93%). Future research should build upon these findings in order to clarify whether similar factors influence implementation among teachers who teach middle and high school, have a greater number of years of experience, and are from more sociodemographically diverse backgrounds. Future research could employ enriched samples to improve racial/ethnic and gender identity diversity in order to capture the broader experience of all teachers. Further, despite the early career status of teachers in this study, participants reported slightly higher averages of burnout than stress. Future research could continue to explore whether other indices of occupational health (e.g., burnout) may also interact with perceptions of feasibility to impact teachers’ implementation of evidence-based programs.

The present study provides some support for the Prosocial Classroom Model and model of factors impacting the implementation of school-based interventions (Domitrovich et al., 2008; Jennings and Greenberg, 2009). However, the interactions between occupational health and perceptions of feasibility found in this study also highlight that those models may be too simplified for the complexity of school-based research. Based on these findings, future research should continue to explore the multitude of program-, teacher- and school-level factors that influence the quality of intervention implementation among teachers with a range of experience and across varying intervention programs.

### 4.6. Implications for practice

Higher dosages of coaching cycles frequently lead to improved implementation fidelity and, ultimately, better student outcomes (Becker et al., 2013; Pas et al., 2022). Therefore, it is important for educators to be motivated to participate in coaching cycles. Results of this study indicate that teachers who perceived PAX GBG + MTP as feasible also participated in more coaching cycles. As such, efforts to increase perceptions of program feasibility may result in greater program dosage. One way to increase perceptions of feasibility is to ensure the program aligns with the school’s core values (Forman et al., 2009). If teachers feel as though the program is a good “fit” to their own goals and philosophies, they are more likely to view the program in a positive way (Forman et al., 2009). Additionally, researchers may consider sharing findings regarding the positive perceptions of PAX GBG + MTP with teachers interested in implementing the program, as teachers respond well to learning new information from other teachers (Forman et al., 2009; Beahm et al., 2021).

Although the results provided no evidence that teachers’ occupational health predicted their dosage and quality of implementation of the PAX GBG + MTP, we are cautious in our interpretation of these findings given that these associations have been found in previous research (e.g., Ransford et al., 2009; Domitrovich et al., 2015). Regardless of its predictive utility for teachers’ implementation of evidence-based programs, experiences of occupational health are salient and meaningful experiences for teachers. When indicating that teachers are suffering from poor occupational health, schools should be motivated to intervene not just because poor occupational health could impact teaching practices and implementation of evidence-based programs but also from a compassionate perspective to alleviate suffering.

The teacher and school demographics included as covariates in this study shed light on who and in what contexts implementation is notably high. Given that teachers of higher grades completed fewer coaching cycles, these teachers may be in need of greater support from coaching staff in order to increase engagement in the program. Although teachers in schools where a higher percentage of students were eligible for FARMS had greater implementation dosage, they simultaneously had lower implementation quality. These findings indicate that these teachers may be in need of additional support, potentially beyond the existing scope of the PAX GBG + MTP program, in order to reach high-quality implementation of PAX GBG + MTP. Taken together, future research should continue to explore teacher and school characteristics that influence both implementation quality and dosage in order to improve student and teacher outcomes.

## 5. Conclusion

The present study advances the field of implementation science in school-based research by investigating the association between teachers’ occupational health and perceptions of program feasibility in relation to the dosage and quality of implementation of two evidence-based programs implemented together. Results provided some support for conceptual models of factors that influence the implementation of school-based interventions (Domitrovich et al., 2015), and highlight the complexity of optimizing implementation in this context. With the growing emphasis on the implementation of evidence-based programs in schools, efforts to scale-up such programs with fidelity should continue to attend to teacher- and school-level contextual factors. This study provides additional empirical evidence of particular characteristics that that may hinder implementation, while identifying potential factors such as program feasibility that may be important for facilitating the implementation of evidence-based programs.

## Data availability statement

The original contributions presented in the study are included in the article, further inquiries can be directed to the corresponding author.

## Ethics statement

The studies involving human participants were reviewed and approved by University of Virginia IRB. The patients/participants provided their written informed consent to participate in this study.

## Author contributions

SB: conceptualization of the paper, formal analysis, and writing—original draft. CB, NI, PT, and JD: conceptualization of the project, funding acquisition, and writing—review and editing. LB and AB: writing—original draft. All authors contributed to the article and approved the submitted version.

## Funding

The research reported here was supported by the Institute of Education Sciences, US Department of Education, through grants R305A190162 and R305A130107 (to the University of Virginia).

## Conflict of interest

The authors declare that the research was conducted in the absence of any commercial or financial relationships that could be construed as a potential conflict of interest.

## Publisher’s note

All claims expressed in this article are solely those of the authors and do not necessarily represent those of their affiliated organizations, or those of the publisher, the editors and the reviewers. Any product that may be evaluated in this article, or claim that may be made by its manufacturer, is not guaranteed or endorsed by the publisher.

## Author disclaimer

The opinions expressed are those of the authors and do not represent views of the US Department of Education.
